# Timing of abortion among adolescent and young women presenting for post-abortion care in Kenya: a cross-sectional analysis of nationally-representative data

**DOI:** 10.1186/s12905-018-0521-4

**Published:** 2018-02-17

**Authors:** Boniface A. Ushie, Chimaraoke O. Izugbara, Michael M. Mutua, Caroline W. Kabiru

**Affiliations:** 1 African Population and Health Research Center, 2nd Floor APHRC Campus, Manga Close Off Kirawa Road, P.O. Box 10787-00100, Nairobi, Kenya; 2 School of Public Health, University of the Witwatersrand, Johannesburg, South Africa

**Keywords:** Abortion, Abortion-related complications, Post-abortion care, Adolescents, Young women, Timing of abortion, Kenya

## Abstract

**Background:**

Complications of unsafe abortion are a leading cause of maternal mortality in sub-Saharan Africa. Adolescents and young women are disproportionately represented among those at risk of these complications. Currently, we know little about the factors associated with young women’s timing of abortion. This study examined the timing of abortion as well as factors influencing it among adolescents and young women aged 12–24 years who sought post-abortion care (PAC) in health facilities in Kenya.

**Methods:**

We draw on data from a cross-sectional study on the magnitude and incidence of induced abortion in Kenya conducted in 2012. The study surveyed women presenting with a diagnosis of incomplete, inevitable, missed, complete, or septic abortion over a one-month data collection period in 328 health facilities (levels 2–6). Survey data, specifically, from adolescents and young women were analyzed to examine their characteristics, the timing of abortion, and the factors associated with the timing of abortion.

**Results:**

One thousand one hundred forty-five adolescents and young women presented for PAC during the data collection period. Eight percent of the women reported a previous induced abortion and 78% were not using a modern method of contraception about the time of conception. Thirty-nine percent of the index abortions occurred after 12 weeks of gestation. A greater proportion of women presenting with late abortions (more than 12 weeks gestational age) (46%) than those presenting with early abortions (33%) presented with severe complications. Controlling for socio-demographic and reproductive history, timing of abortion was significantly associated with place of residence (marginal), education, parity, clinical stage of abortion and level of severity.

**Conclusions:**

Late-term abortions were substantial, and may have contributed substantially to the high proportion of women with post-abortion complications. Efforts to reduce the severity of abortion-related morbidities and mortality must target young women, particularly those living in rural and other remote areas. Interventions to reduce unintended pregnancies in this population are also urgently needed to improve early pregnancy detection and timely care seeking.

## Background

Globally, pregnancy termination resulting from unwanted and/or mistimed conception is a major maternal health issue, especially in settings where abortion laws are restrictive. The health risks posed by pregnancy termination are increased if timely access to abortion care is not secured, leading to adverse outcomes [1]. In many countries in sub-Saharan Africa, safe abortion is restricted. In such contexts, many women, especially young women in need of pregnancy termination resort to unsafe abortion procedures [2], and/or delay the decision to seek an abortion resulting in late-term abortions, which are associated with significant risks including fatalities [3].

Adolescents and young women constitute the bulk of women who experience problems resulting from unsafe abortion [4] due to their high risk for unintended pregnancies and lack of access to safe abortion services [5–9]. The large proportion of unintended pregnancies in this age group is driven, in part, by their limited power to negotiate safe sexual activity and contraceptive use, as well as inadequate access to sexual and reproductive health information and services [10, 11]. Further, adolescents often lack the financial and other resources to seek appropriate care from well-equipped clinics, and hence, resort to poorly equipped low-cost facilities or self-induced abortion [10, 11]. The implication is that their pathway to proper care is often long and winding. Efforts to understand the timing of abortion and the factors that shape timing decisions among adolescents and young women are limited by the lack of reliable data. Yet, such information is key to the development and delivery of innovative interventions. In this study, we investigated the timing of abortion and associated factors among adolescents and young women 12–24 presenting for post-abortion care (PAC) in Kenya in a nationally-representative sample of health facilities.

Kenya offers an interesting context for understanding the timing of abortion and associated factors among adolescents and young women. Although the Kenyan Constitution offers potential for increasing women’s access to safe abortion [12], societal attitudes towards young women who procure abortion remain largely negative [13]. While access barriers to safe abortion remain unaddressed, young women will not only delay the request for termination but also use the options that are within their reach while maintaining secrecy to reduce the number of people who become aware that they have had an abortion to forestall stigmatization [12, 13], a practice that persistently leads to negative outcomes. In the current study, we present the socio-demographic and reproductive characteristics, describe the characteristics of the pregnancy for which they were seeking PAC and the severity of the complications they presented with. We also examine the factors associated with the timing of abortion among them.

## Methods

### Data and procedures

This study draws on secondary data from a cross-sectional survey on the magnitude and incidence of induced abortion in Kenya conducted in 2012 [14]. The survey was conducted in 328 nationally-representative health facilities (out of 2838 levels 2–6 facilities). Health facilities were selected using stratified random sampling. Stratification was based on the Kenya Essential Package for Health (KEPH) classification of six levels of preventive and curative health services, type of ownership (government or private/non-governmental), and geographical region. All sampled facilities (including public, private for profit and not-for-profit) had the capacity to provide PAC services. In each facility, health providers were trained to collect data from all women presenting for PAC with a diagnosis of incomplete, inevitable, missed and complete abortion. Data were collected over a thirty-day period (April–May) using an abortion case capture form, which elicited information on socio-demographic information, reproductive history, clinical history and physical examination findings, and diagnosis.

During the one-month data collection period, 2625 women presented for PAC, while 528 presented for termination of pregnancies (total = 3153). Of the 2625 women who sought PAC, 1408 were adolescents and young women aged 12–24 years. We excluded data from 263 young women who presented for abortion induction or PAC after 24 weeks of gestation. The analytical sample is therefore based on data from 1145 adolescents and young women aged 12–24 years who sought PAC services.

### Variables

The primary outcome variable assessed was the timing of abortion measured as the gestational age at abortion. Gestational age at abortion was assessed as a binary variable indicating whether the gestational age of the index pregnancy was less than or equal to 12 weeks or greater than 12 weeks. The study only considers these two cases and therefore the outcome variable is binary, focusing on the later abortion as the outcome and early abortion as the reference group. The primary explanatory variables are the nature of index abortion (spontaneous or induced); previous live births (no children or one/more); previous induced abortion (none or one/more); use of contraception at the time of the index pregnancy (yes or no), pregnancy intendedness (then, later, not at all), number of children (none vs one/more), and previous miscarriage (yes vs no). The socio-demographic characteristics of interest included age (measured as a categorical variable with two categories: 12–19 years and 20–24 years); marital status (never married, married/living together, separated/divorced/widowed); residence (rural or urban); education (no formal school, primary, secondary, post-secondary); occupation (farmer/unskilled, skilled/clerical, student, unemployed/housewife/other) and region (Central and Nairobi, Coast/North Eastern, Eastern, Nyanza and Western, Rift Valley). We also investigated the association between the timing of abortion and the severity of PAC complications. Severity of PAC complications was assessed using an adaptation of the Rees et al.'s [15] three-level abortion complication severity as follows: severe (body temperature of > 37.9 °C, organ or system failure, generalized peritonitis, pulse > 119 beats/min, evidence of foreign body or mechanical injury, sepsis, shock, and/or tetanus); moderate (body temperature between 37.3–37.9 °C, adnexal or abdominal tenderness, localized peritonitis, and/or offensive products of conception) or low (all other cases).

### Analyses

We computed descriptive statistics of the respondents’ socio-demographic characteristics, reproductive and clinical histories, as well as physical examination findings and diagnosis. We then conducted bivariate analyses to examine factors associated with gestational age at abortion, including the severity of PAC complications. Finally, we used multivariable logistic regression analysis to assess the factors associated with gestational age at abortion. In fitting the logistic regression models, we used a binary dependent variable that identified at which trimester an adolescent or young woman procured the abortion that resulted in the complications for which they were being treated. The explanatory variables in the logistic model were women’s demographic, socio-economic and reproductive characteristics. First, we fitted bivariate models to estimate the associations between the dependent variable and each explanatory variable. Second, we fitted a model with the socio-demographic variable before fitting a model with all independent variables.

## Results

Respondents’ socio-demographic characteristics and reproductive histories by by timing of abortion are summarized in Table 1. Thirty-seven percent of the women were presenting for PAC for abortions that occurred after more than 12 weeks of gestational age. Thirty-four percent of young care-seekers were aged 12–19 years. A greater proportion of young women presenting for PAC for late term abortion (≥12 weeks of gestation) (38%) than those presenting for abortions at less than 12 weeks gestational age (33%) were aged 12–19 years. About half (47%) of these care-seekers had never been married and 53% had secondary or higher education. Majority (62%) of the respondents lived in rural areas. Most of the respondents (60%) had never had a live birth. Forty percent of the respondents reported that they were seeking PAC for an induced abortion; 8% reported a previous induced abortion. Majority of the respondents (78%) were not using any form of modern contraceptives at the time of conception of the index pregnancy for which PAC was sought. Sixty-one percent of respondents stated that the index pregnancy was unintended (not wanted at all or mistimed).

**Table 1:** Respondents’ socio-demographic and reproductive characteristics by age group and residence

Variable	Categories	All	Timing of abortion^a^	
		(*N* = 1145)	≤12 weeks (*n* = 717)	> 12 weeks (*n* = 426)
Socio-demographics:				
Age	12–19 (*n* = 335)	34.2	32.3	37.5
	20–24 (*n* = 810)	65.8	67.7	62.5
Marital status	Never married (*n* = 549)	46.9	47.5	45.7
	Married/Living together (*n* = 561)	48.7	48.3	49.8
	Separated/ Divorced/Widowed (*n* = 33)	4.2	4.1	4.4
Religion	Roman Catholic (*n* = 232)	19.3	18.4	21
	Other Christian (*n* = 798)	69.0	66.5	72.5
	Muslim (*n* = 98)	9.2	11.7	5.4
	Other religion (*n* = 14)	2.3	3.2	1
Education	No education (*n* = 57)	7.1	8.1	5.7
	Primary (*n* = 425)	39.7	36	46
	Secondary (*n* = 471)	43.2	43.3	42.3
	Post-secondary (*n* = 189)	9.9	12.5	5.9
Occupation	Farmer/unskilled (*n* = 187)	20.3	20.1	19.8
	Skilled/clerical (*n* = 181)	12.4	11.7	13.7
	Student (*n* = 321)	23.6	22.4	25.7
	Unemployed/Housewife/Other (*n* = 453)	43.6	45.6	40.8
Region	Central & Nairobi (*n* = 304)	20.2	22.1	17.6
	Coast & N. Eastern (*n* = 175)	14.3	16.6	10.9
	Eastern (*n* = 156)	7.0	6.1	8.5
	Nyanza & Western (*n* = 290)	36.9	34.5	41.2
	Rift valley (*n* = 220)	21.5	20.7	21.7
Nature of index abortion	Spontaneous (*n* = 747)	59.9	45.1	67.6
	Induced (*n* = 398)	40.1	54.9	32.4
Reproductive history:				
Previous live births	None (*n* = 706)	60.2	58.4	62.6
	1 or more (*n* = 435)	39.8	41.6	37.4
Previous abortions	None (*n* = 1069)	92.1	93.3	90.1
	1+ (*n* = 71)	7.7	6.4	9.9
Contraceptive use	Yes (*n* = 249)	22.4	24.9	18.7
	No (*n* = 896)	77.6	75.1	81.3
Pregnancy desired	Then (*n* = 421)	35.4	34.9	36.5
	Later (*n* = 302)	28.1	27	29
	Not at all (*n* = 376)	32.8	33.8	31.6
Number of children	None (*n* = 724)	61.3	59.5	63.7
	1 or more (*n* = 417)	38.7	40.5	36.3
Previous miscarriage	Yes (*n* = 130)	11.8	12.7	10.6
	No (*n* = 1015)	88.2	87.3	89.4

^a^2 cases with missing timing data were excluded

**Fig. 1:**
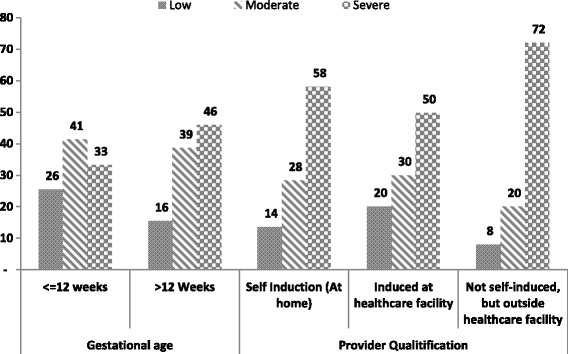
Presents severity of complications. Level of Complications by Gestational Age at Abortion by the timing of abortion among young women who sought post abortion care. Gestational age refers to the age of pregnancy at the time of termination

Table 2 highlights the characteristics of the pregnancy for which respondents were seeking post-abortion care. Clinical diagnosis data showed that 68% of young women seeking PAC services presented with an incomplete abortion. One in five women reported that the abortion was self-induced, while two in five women reported that they first received abortion care from untrained personnel. Thirty-eight percent of PAC clients presented with severe complications. A greater proportion of women presenting with late abortions (more than 12 weeks gestational age) (46%) than those presenting with early abortions (33%) presented with severe complications (Fig 1).

**Table 2:** Characteristics of pregnancy for which PAC was sought by timing of abortion

Reproductive characteristics	%	Timing of abortion^a^			
	(*N* = 1145)	≤12 weeks (*n* = 717)	> 12 weeks (*n* = 426)	Pr (χ2_c_ > χ2)	Sign
Clinical abortion stage					
Inevitable Abortion (*n* = 120)	9.9	6.9	14.7	0.242	
Incomplete Abortion (*n* = 842)	67.6	69.5	64.4		
Missed Abortion (*n* = 37)	2.7	2.8	2.6		
Complete Abortion (*n* = 146)	19.8	20.8	18.4		
Nature of index abortion					
Spontaneous (*n* = 747)	59.9	61.8	57.5	0.233	
Induced (*n* = 398)	40.1	38.2	42.5		
Facility of first care					
Self-Induction (*n* = 57)	19.8	17.0	24.2	0.516	
Trained personnel (*n* = 210)	40.7	43.5	37.5		
cUntrained personnel (*n* = 131)	39.6	39.5	38.2		
Severity of PAC complication					
Low (*n* = 299)	21.5	25.5	15.5	0.034	*
Moderate (*n* = 482)	40.2	41.3	38.6		
Severe (*n* = 364)	38.3	33.2	45.9		

^a^2 cases with missing timing data were excluded

*** *p* < 0.01; ** *p* < 0.05; * *p* < 0.1; † *p* > =0.1

Table 3 summarizes results of bivariate and multivariable logistic regression analyses to assess factors associated with the timing of abortion. At bivariate level, age, marital status, place of residence (marginally), education, parity, contraceptive use, pregnancy intendedness, and initial care were significantly associated with the timing of abortion. However, in the multiple regression model including socio-demographic variables only (Model 1), only education was significantly associated with the timing of abortion. Adolescents and young women with post-secondary education were significantly less likely to present for PAC of a second trimester abortion compared with those with no education. When pregnancy-related variables were included, only area of residence was significantly associated with the timing of abortion with residents of rural areas being twice as likely to present for PAC of a second trimester abortion. None of the pregnancy-related variables were associated with the timing of abortion. At model 3, when only variables with a *p*-value of 0.2 from model 2 are included, severity of complications, clinical abortion stage, level of education and previous live birth were associated with timing of abortion.

**Table 3:** Logistic regression models of correlates of second trimester abortion at ≥12 weeks gestational age among girls and young women (12–24 years)

	Model 0				Model 1				Model 2				Model 3			
	95% Conf. interval				95% Conf. interval				95% Conf. interval				95% Conf. interval			
	Odds Ratio	Lower	Upper	Sign	Odds Ratio	Lower	Upper	Sign	Odds Ratio	Lower	Upper	Sign	Odds Ratio	Lower	Upper	Sign
Age																
12–19 (Ref)																
20–24	0.584	0.449	0.758	***	0.780	0.529	1.151		0.972	0.653	1.447					
Marital status																
Single/never married (Ref)																
Married/Cohabitation	0.652	0.505	0.842	**	0.918	0.577	1.462		1.037	0.565	1.904		–	–	–	
Sep/Wid/Div	0.675	0.291	1.564		1.008	0.374	2.714		1.178	0.431	3.221		–	–	–	
Residence																
Urban (Ref)																
Rural	0.754	0.552	1.031	†	1.149	0.771	1.713		1.433	0.945	2.171	†	1.439	0.973	2.130	†
Education																
None (Ref)																
Primary	0.808	0.607	1.075		0.878	0.560	1.378		1.049	0.499	2.205		1.066	0.553	2.054	
Secondary	0.618	0.415	0.920	*	0.697	0.446	1.089		0.809	0.374	1.748		0.829	0.428	1.606	
Post-secondary	0.297	0.164	0.536	***	0.368	0.187	0.725	**	0.423	0.160	1.121	†	0.429	0.189	0.973	*
Previous live birth																
None (Ref)																
One or more	0.570	0.426	0.764	***					0.630	0.400	0.990	*	0.653	0.435	0.983	*
Contraceptive use																
Using modern (Ref)																
Not using	0.684	0.519	0.901	**					1.226	0.697	2.157		–	–	–	
Pregnancy desired																
Wanted then (Ref)																
Wanted latter	0.677	0.444	1.033	†					1.097	0.634	1.899		–	–	–	
Never wanted	0.591	0.407	0.856	**					0.711	0.391	1.294		–	–	–	
Clinical abortion stage																
Missed Abortion (Ref)																
I nevitable Abortion	1.340	0.766	2.345						1.057	0.370	3.023		0.982	0.387	2.493	
Incomplete Abortion	0.585	0.440	0.778	***					0.368	0.139	0.970	*	0.385	0.176	0.842	*
Complete Abortion	0.559	0.343	0.912	*					0.313	0.102	0.955	*	0.365	0.138	0.967	*
Facility of first care																
Spontaneous (Ref)																
Self-induction	1.003	0.512	1.964						1.323	0.616	2.840		–	–	–	
Trained personnel	0.679	0.348	1.323						0.755	0.408	1.399		–	–	–	
Untrained personnel	0.606	0.401	0.917	*					1.178	0.683	2.030		–	–	–	
Severity of PAC complication																
Low (Ref)																
Moderate	0.590	0.430	0.811	**					1.384	0.826	2.320		1.407	0.833	2.374	
Severe	0.874	0.639	1.197						2.555	1.546	4.222	***	2.331	1.482	3.668	***

†*p* < .10; *p < .05; **p < .01; ***p < .001

## Discussion

Late term abortion, whether induced or spontaneous, has the tendency to result in complications. The severity of complications resulting from abortion among young women can be reduced if appropriate abortion care is sought in time. In this study, we drew on nationally-representative data to examine the timing of abortion and factors associated with timing among adolescents and young women (12–24 years) presenting for post-abortion care in Kenya. More than one in three young women presented for abortion-related care for abortions that occurred later than the 12th week of gestation. Young women presenting late were more likely to have severe complications.

Although adolescents (12–19 years) comprised just over a third of respondents, they were more likely to present for PAC following a second trimester abortion. In contexts where abortion is illegal, late term induced abortion may be directly related to the difficulties of procuring an abortion. However, where abortion services are available and legal, studies have found delayed timing of abortion to be associated with stigmatization, which could be both at the community and health facility levels [22]. Moreover, issues of the legality of abortion also play a significant role in timing decisions, among other barriers including costs and availability of services.

Our study shows some marginal associations between place of residence and timing of abortion. Adolescents from rural areas were more likely to present for PAC with second-trimester abortion compared to their urban counterparts. This finding is corroborated by previous studies (e.g [22]) which show how women in rural areas were at higher risk of seeking late abortion, despite the availability of first-trimester abortion. Moreover, studies have shown that rural dwellers have poorer access to good health facilities [16–19] and may, therefore, delay seeking appropriate abortion care, which is more likely to result in complications. These factors are therefore likely to put rural dwellers at greater risk for negative sexual and reproductive health outcomes associated with unsafe abortions [20, 21].

Close to two-thirds of induced abortions were either self-induced or initially induced by other persons outside formal healthcare facilities. We found 42% of all late term abortion was induced. Expectedly, therefore, nearly four out of five young women presented with moderate or severe complications. As Mulat et al. [22] have noted, late term induced abortion could be attributed to difficulties in early recognition of pregnancy among adolescents, irregularity of menstrual cycles (which is associated with poor pregnancy signs recognition because makes it difficult to recognize when one has become pregnant), and other logistical problems. In our study we have found adolescents with higher level of education less likely to seek late term abortion. Studies have shown sexuality education to be a key factor in adolescent decision-making regarding their sexual and reproductive health. Furthermore, a substantial proportion of spontaneous abortion was observed in this study, and this may point to the need for increased and improved support to women on caring for pregnancies. Previous studies have found that poor support and access to care for pregnancy can directly influence late term spontaneous abortion among other factors such as adverse drug effects and alcohol use [23].

Providing safe abortion care as one of the strategies for improving maternal health has to go beyond the mere availability and affordability of the services, but must deal with advocacy and sexuality education. Ensuring access to and enhancing utilization of youth-friendly emergency obstetric services may be a critical element of the efforts to reduce the incidence and magnitude of unsafe abortions and the complications that result from them. Overall, however, the low use of contraception among young women in this study suggests that preventing unintended pregnancies among adolescents and young women may be key to reducing, first, unwanted pregnancies, and consequently, induced abortion [24]. Also, improving access to quality and safe second trimester abortion services, and increasing counselling services and logistical support to adolescent and young women may be helpful.

In particular, efforts must be made to reach adolescents and those living in rural areas. This study has three key limitations: first, apart from place of residence, majority of the factors assessed were not significant predictors of the timing of abortion; for example, contraception was not significant after adjustment, which could be due to the temporality of its usage. This lack of association might reflect the limited scope of measures or lack of variability. Second, data collected were sensitive in nature and may therefore be subject to reporting bias. However, most of the data used in this paper are observational and reported by the physician, a technique that significantly reduces reporting bias. Third, this being a facility-based study we might have missed out a substantial proportion of young women who either did not seek post-abortion care or sought care in facilities that typically do not provide post-abortion care services and were therefore excluded from the sample. However, despite these limitations, this paper draws on rich data from post-abortion care seekers in a nationally representative sample of health facilities and highlights important factors associated with the timing of abortion among adolescents and young women aged 12–24 years.

## Conclusions

Complications resulting from unsafe abortion among young and adolescent women have far-reaching consequences for maternal health especially in settings with restrictive abortion laws. Our study indicates that a significant proportion of young women presenting for PAC in Kenyan health facilities present for care following a second trimester abortion and that these late-term abortions are associated with a higher likelihood of complications than abortions occurring in the first trimester. Efforts to reduce unsafe abortion and associated complications must target young women; particularly adolescents aged 12–19 years and those living in rural areas. Interventions to reduce unintended pregnancies are also urgently needed. Preventive measures could include more targeted policies geared towards availing comprehensive sexual and reproductive health information and services to adolescents and young women. The role of effective youth-friendly contraceptive service provision and post-abortion care must be emphasized in order to reduce risks of unintended pregnancies.

## Abbreviations

KEMRI: Kenya Medical Research Institute
KEPH: Kenya Essential Package for Health
PAC: Post-abortion care
